# Excellence in Clinical Practice Through the Evidence-Based Medicine

**DOI:** 10.5812/cardiovascmed.5417

**Published:** 2012-11-01

**Authors:** Majid Haghjoo

**Affiliations:** 1 Cardiac Electrophysiology Research Center, Rajaie Cardiovascular Medical and Research Center, Tehran University of Medical Sciences, Tehran, IR Iran

**Keywords:** Cardiology, Medicine

*“There is within medicine, somewhere beneath the pessimism and discouragement resulting from the disarray of the health care system and its stupendous cost, an undercurrent of almost outrageous optimism about what may lie ahead for the treatment of human disease if only we can keep learning”*

*- Lewis Thomas (1)*

Traditionally, clinicians have been credited with clinical wisdom according to their skills in making a diagnosis and prescribing a treatment. The advent of major investments in biomedical research, leading to new and better tests and treatments, has spurred the development of critical appraisal of the medical literature and evidence-based medicine (2), and application of current best evidence from healthcare research is now an expected adjunct to clinical wisdom.

Evidence-based medicine, whose philosophical origins extend back to mid-19th century Paris and earlier, is the conscientious, explicit and judicious use of current best evidence in making decisions about the care of individual patients (3). The practice of evidence-based medicine means integrating individual clinical expertise with the best available external clinical evidence from systematic research. Increased expertise is reflected in many ways, but especially in more effective and efficient diagnosis and in the more thoughtful identification and compassionate use of individual patients' predicaments, rights, and preferences in making clinical decisions about their care. By best available external clinical evidence we mean clinically relevant research, often from the basic sciences of medicine, but especially from patient centered clinical research into the accuracy and precision of diagnostic tests (including the clinical examination), the power of prognostic markers, and the efficacy and safety of therapeutic, rehabilitative, and preventive regimens. 

Evidence-based medicine initially focused mainly on determining the best research evidence relevant to a clinical problem or decision and applying that evidence to resolve the issue. Subsequent versions of evidence-based decision making have emphasized that research evidence alone is not an adequate guide to action. Rather, clinicians must apply their expertise to assess the patient's problem and must also incorporate the research evidence and the patient's preferences or values before making a management recommendation (4).

Similar to other fields, specialists in the field of cardiovascular medicine are faced with the dilemma that many clinical questions in their daily practice to do not have universally agreed answers, but patients increasingly demand the ‘best practice’ from their doctors. In addition time pressures mean that clinicians are unable to keep up with the full spectrum of published research and current resources that collate evidence for clinicians. In our scrupulous scrutiny to provide up-to-date and practical data, our fully online and peer-reviewed journal, Research in Cardiovascular Medicine (RCVM), was founded in 2011. RCVM, the official journal of Rajaie Cardiovascular Medical and Research Center, is dedicated to the clinical and basic researches in the field of cardiovascular medicine. This authoritative medical journal publishes peer-reviewed articles on all aspects of cardiovascular disease, including original articles, systematic reviews, meta-analyses, narrative reviews, short articles, letters, case reports, and interesting images. This journal accepts the articles only from an online submission system. This online editorial system is prepared for manuscript submission, peer reviewing and tracking replications. Our system is customized with training and step-by-step tips in order to provide a very convenient service for authors, reviewers and editors. By available tracking service, authors can track the review process of their submitted manuscript. RCVM is an open-access journal, in order to provide appropriate updated scientific information in the field of cardiovascular medicine for the relevant readers, all around the world and is published in co-operation with Tehran University of Medical Sciences. I hope RCVM meet the high expectations of our readers by providing evidence based data in the field of cardiovascular medicine.
